# Supplementary material for: Intensive versus conservative glycemic control in patients undergoing coronary artery bypass graft surgery: A protocol for systematic review of randomised controlled trials
Source: PLoS One. 2022 Oct 18;17(10):e0276228. doi: 10.1371/journal.pone.0276228 (PMC9578579; doi:10.1371/journal.pone.0276228)
Supplement: S1 Table — (DOCX) [file pone.0276228.s002.docx]

**S1 Table: Draft of Data Extraction Template**

| **Table 1 Data extraction template** | |
| --- | --- |
| **Study characteristics** | Journal, language, first author, publication date, country, publication status, study design and setting. |
| **Population** | Gender, age, body mass index (BMI), the duration of diabetes mellitus (if present), the duration of coronary heart disease, the duration of hypertension (if present), smoking status, ejection fraction (EF), the type of CABG (on-pump or off-pump), operative status (elective or other status), cardiopulmonary bypass time (CPB time), cross-clamp time |
| **Interventions** | Insulin, glucose-insulin-potassium (GIK) solution or hyperinsulinemic normoglycemic clamp (modified GIK solution) |
| **Comparators of two groups** | The level of blood glucose in each group, the number of patients in each group |
| **Outcomes** | Postoperative mortality, the duration of mechanical ventilation in ICU, the incidence of postoperative myocardial infarction (MI), the incidence of postoperative atrial fibrillation (AF), blood product transfusion (the type and volume), the rate of rehospitalization, the rate of cerebrovascular accident, the rate of significant postoperative bleeding, the rate of infection, the incidence of acute kidney failure (AKF), hospital and ICU lengths of stay (LOS). |
