# Supplementary material for: Intensive versus conservative glycemic control in patients undergoing coronary artery bypass graft surgery: A protocol for systematic review of randomised controlled trials
Source: PLoS One. 2022 Oct 18;17(10):e0276228. doi: 10.1371/journal.pone.0276228 (PMC9578579; doi:10.1371/journal.pone.0276228)
Supplement: S1 File — (DOCX) [file pone.0276228.s003.docx]

**S1 File: Search Strategies**

**The detailed search strategies of the four databases:**

| **Search strategy of PubMed** | |
| --- | --- |
| #1 | (“Glycemic Control”[Mesh]) OR (Control, Glycemic) OR (Blood Glucose Control) OR (Control, Blood Glucose) OR (Glucose Control, Blood) |
| #2 | (“Coronary Artery Bypass”[Mesh]) OR (Artery Bypass, Coronary) OR (Artery Bypasses, Coronary) OR (Bypasses, Coronary Artery) OR (Coronary Artery Bypasses) OR (Coronary Artery Bypass Surgery) OR (Bypass, Coronary Artery) OR (Aortocoronary Bypass) OR (Aortocoronary Bypasses) OR (Bypass, Aortocoronary) OR (Bypasses, Aortocoronary) OR（Bypass Surgery, Coronary Artery）OR (Coronary Artery Bypass Grafting) |
| #3 | (“Coronary Artery Bypass, Off-Pump”[Mesh]) OR (Coronary Artery Bypass, Off Pump) OR (Coronary Artery Bypass, Beating Heart) OR (Off-Pump Coronary Artery Bypass) OR (Off Pump Coronary Artery Bypass) OR (Beating Heart Coronary Artery Bypass) |
| #4 | #1 AND (#2 OR #3) |

| **Search strategy of Embase.com** | |
| --- | --- |
| #1 | 'coronary artery bypass'/exp/mj OR (artery AND bypass, AND coronary) OR (artery AND bypasses, AND coronary) OR (bypasses, AND coronary AND artery) OR (coronary AND artery AND bypasses) OR (coronary AND artery AND bypass AND surgery) OR (bypass, AND coronary AND artery) OR (aortocoronary AND bypass) OR (aortocoronary AND bypasses) OR (bypass, AND aortocoronary) OR (bypasses, AND aortocoronary) OR (bypass AND surgery, AND coronary AND artery) OR (coronary AND artery AND bypass AND grafting) |
| #2 | 'coronary artery bypass, off-pump'/exp/mj OR (coronary AND artery AND bypass, AND off AND pump) OR (coronary AND artery AND bypass, AND beating AND heart) OR ('off pump' AND coronary AND artery AND bypass) OR (off AND pump AND coronary AND artery AND bypass) OR (beating AND heart AND coronary AND artery AND bypass) |
| #3 | #1 OR #2 |
| #4 | 'glycemic control'/exp/mj OR (control, AND glycemic) OR (blood AND glucose AND control) OR (control, AND blood AND glucose) OR (glucose AND control, AND blood) |
| #5 | #3 AND #4 |

| **Search strategy of Cochrane Library** | |
| --- | --- |
| #1 | MeSH descriptor: [Glycemic Control] explode all trees |
| #2 | MeSH descriptor: [Coronary Artery Bypass] explode all trees |
| #3 | MeSH descriptor: [Coronary Artery Bypass, Off-Pump] explode all trees |
| #4 | (Artery Bypass, Coronary):ti,ab,kw OR (Artery Bypasses, Coronary):ti,ab,kw OR (Bypasses, Coronary Artery):ti,ab,kw OR (Coronary Artery Bypasses):ti,ab,kw OR (Coronary Artery Bypass Surgery):ti,ab,kw (Word variations have been searched) |
| #5 | (Bypass, Coronary Artery):ti,ab,kw OR (Aortocoronary Bypass):ti,ab,kw OR (Aortocoronary Bypasses):ti,ab,kw OR (Bypass, Aortocoronary):ti,ab,kw OR (Bypasses, Aortocoronary):ti,ab,kw (Word variations have been searched) |
| #6 | (Bypass Surgery, Coronary Artery):ti,ab,kw OR (Coronary Artery Bypass Grafting):ti,ab,kw OR (Coronary Artery Bypass, Off Pump):ti,ab,kw OR (Coronary Artery Bypass, Beating Heart):ti,ab,kw OR (Off-Pump Coronary Artery Bypass):ti,ab,kw (Word variations have been searched) |
| #7 | (Off Pump Coronary Artery Bypass):ti,ab,kw OR (Beating Heart Coronary Artery Bypass):ti,ab,kw (Word variations have been searched) |
| #8 | (Control, Glycemic):ti,ab,kw OR (Blood Glucose Control):ti,ab,kw OR (Control, Blood Glucose):ti,ab,kw OR (Glucose Control, Blood):ti,ab,kw (Word variations have been searched) |
| #9 | #1 OR #8 |
| #10 | #2 OR #3 OR #4 OR #5 OR #6 OR #7 |
| #11 | #9 AND #10 |

| **Search strategy of Web of Science** | |
| --- | --- |
| #1 | TS= (“Coronary Artery Bypass” OR “Artery Bypass, Coronary” OR “Artery Bypasses, Coronary” OR “Bypasses, Coronary Artery” OR “Coronary Artery Bypasses” OR “Coronary Artery Bypass Surgery” OR “Bypass, Coronary Artery” OR “Aortocoronary Bypass” OR “Aortocoronary Bypasses” OR “Bypass, Aortocoronary” OR “Bypasses, Aortocoronary” OR “Bypass Surgery, Coronary Artery” OR “Coronary Artery Bypass Grafting”) |
| #2 | TS= (“Coronary Artery Bypass, Off-Pump” OR “Coronary Artery Bypass, Off Pump” OR “Coronary Artery Bypass, Beating Heart” OR “Off-Pump Coronary Artery Bypass” OR “Off Pump Coronary Artery Bypass” OR “Beating Heart Coronary Artery Bypass”) |
| #3 | #1 OR #2 |
| #4 | TS= (“Glycemic Control” OR “Control, Glycemic” OR “Blood Glucose Control” OR “Control, Blood Glucose” OR “Glucose Control, Blood”) |
| #5 | #3 AND #4 |

**The detailed search strategies of two trial registries:**

1. **Search strategy of ClinicalTrials.gov as follows:**

( glycemic control OR control, glycemic OR blood glucose control OR control, blood glucose OR glucose control, blood ) AND ( Coronary Artery Bypass OR Artery Bypass, Coronary OR Artery Bypasses, Coronary OR Bypasses, Coronary Artery OR Coronary Artery Bypasses OR Coronary Artery Bypass Surgery OR Bypass, Coronary Artery OR Aortocoronary Bypass OR Aortocoronary Bypasses OR Bypass, Aortocoronary OR Bypasses, Aortocoronary OR Bypass Surgery, Coronary Artery OR Coronary Artery Bypass Grafting OR Coronary Artery Bypass, Off-Pump OR Coronary Artery Bypass, Off Pump OR Coronary Artery Bypass, Beating Heart OR Off-Pump Coronary Artery Bypass OR Off Pump Coronary Artery Bypass OR Beating Heart Coronary Artery Bypass )

1. **International Clinal Trials Registry Platform (ICTRP) was searched through Cochrane Library.**
