# Supplementary material for: Intensive versus conservative glycemic control in patients undergoing coronary artery bypass graft surgery: A protocol for systematic review of randomised controlled trials
Source: PLoS One. 2022 Oct 18;17(10):e0276228. doi: 10.1371/journal.pone.0276228 (PMC9578579; doi:10.1371/journal.pone.0276228)
Supplement: S2 File — (DOCX) [file pone.0276228.s004.docx]

**S2 File: Study Eligibility Form**

**Glycemic control in patients undergoing coronary artery bypass graft surgery**

**Study eligibility form**

**Study ID:**

**Screener:**

**Type of study**

Q1. Is the study described as a randomised controlled trial?

Yes Unclear No

Go to the nest question Exclude

**Participants in the study**

Q2. Did the participants in the study undergo coronary artery bypass graft surgery?

Yes Unclear No

Go to the nest question Exclude

**Interventions in the study**

Q3. Were the participants divide into at least two group based on blood glucose levels?

Yes Unclear No

Go to the nest question Exclude

Q4. Was glycemic control implemented during surgery?

Yes Unclear No

Go to the nest question Exclude

**Comparisons in the study**

Q5. Did the participants in control group undergo blood glucose management?

Yes Unclear No

Go to the nest question Exclude

**Outcomes in the study**

Q6. Did the study report any outcome indicator of this systematic review?

Yes Unclear No

Go to the nest question Exclude

**Final decision:**

Include □ Unclear □ Exclude □
