# Supplementary figures and images for: Intensive versus conservative glycemic control in patients undergoing coronary artery bypass graft surgery: A protocol for systematic review of randomised controlled trials
Source: PLoS One. 2022 Oct 18;17(10):e0276228. doi: 10.1371/journal.pone.0276228 (PMC9578579; doi:10.1371/journal.pone.0276228)

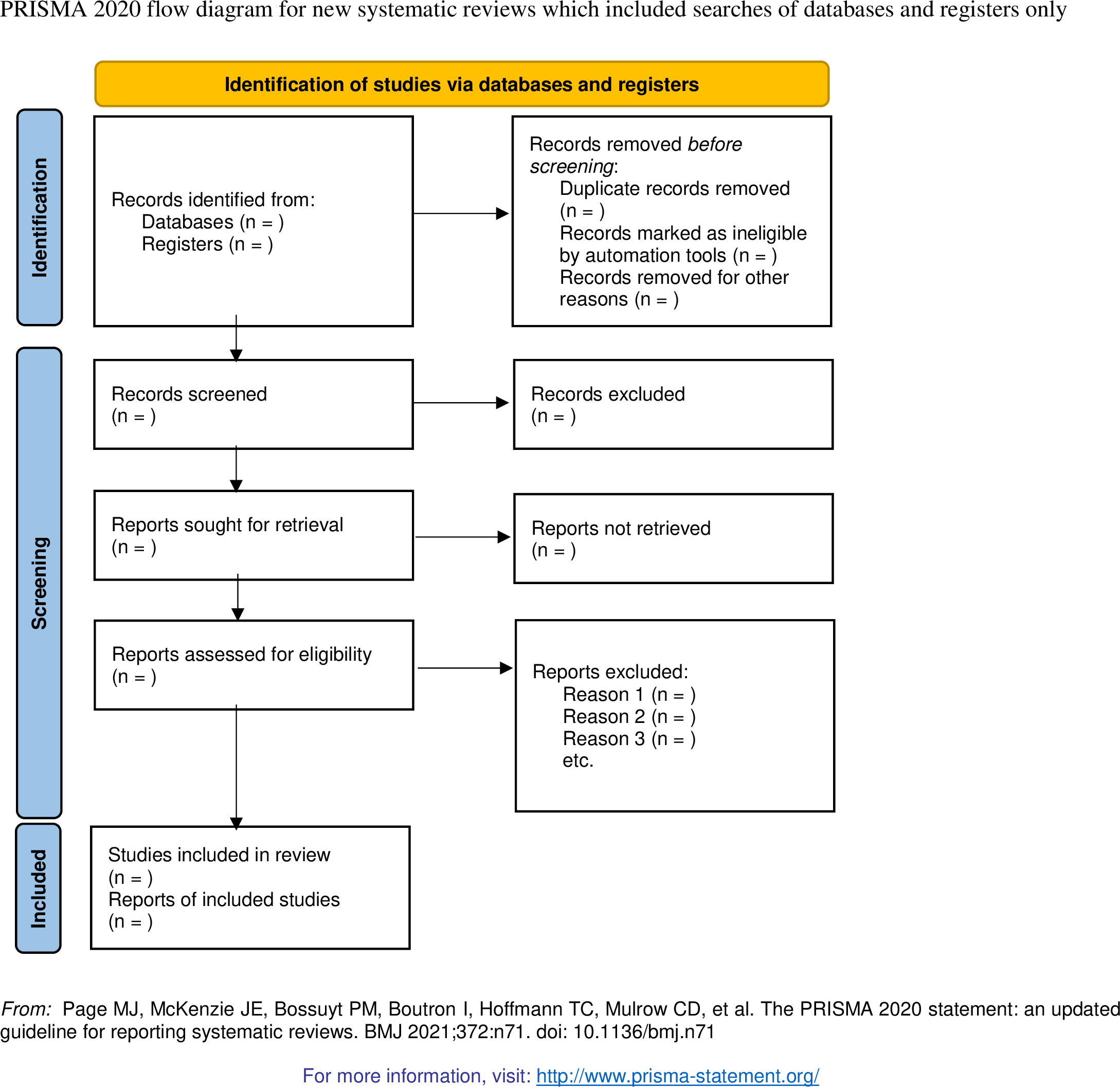

Supplement: S3 File — (TIF) [file pone.0276228.s005.tif]
