# Supplementary material for: Intensive versus conservative glycemic control in patients undergoing coronary artery bypass graft surgery: A protocol for systematic review of randomised controlled trials
Source: PLoS One. 2022 Oct 18;17(10):e0276228. doi: 10.1371/journal.pone.0276228 (PMC9578579; doi:10.1371/journal.pone.0276228)
Supplement: S4 File — (DOCX) [file pone.0276228.s006.docx]

**S4 File: Assessment of Evidence Quality by GRADE approach**

**Author(s):**
**Date:** 2022-07-03
**Question:** Intensive vs Conservative for postoperative outcomes of patients undergoing CABG
**Settings:**
**Bibliography:**  intensive glycemic control versus conservative glycemic control for postoperative outcomes of patients undergoing CABG. Cochrane Database of Systematic Reviews [Year], Issue [Issue].

| **Quality assessment** | | | | | | | **No of patients** | | **Effect** | | **Quality** | **Importance** |  |
| --- | --- | --- | --- | --- | --- | --- | --- | --- | --- | --- | --- | --- | --- |
|  |  |  |  |  |  |  |  |  |  |  |  |  |  |
| **No of studies** | **Design** | **Risk of bias** | **Inconsistency** | **Indirectness** | **Imprecision** | **Other considerations** | **Intensive** | **Conservative** | **Relative (95% CI)** | **Absolute** |  |  |  |
| **Incidence of postoperative MI (follow-up 0-12 weeks; assessed with: ECG or myocardial enzyme)** | | | | | | | | | | | | |  |
| Number of eligible studies reported the outcome | randomised trials |  |  |  |  |  | A^1^  (B%) | B^1^ (2.8%) | RR  (95%CI) | C^2^ fewer per 1000 |  |  |  |
|  |  |  |  |  |  |  |  |  |  | D^2^ fewer per 1000 |  |  |  |

^1^ It stands for some ratio.

^2^ It represents a specific number.
