# Supplementary material for: Intensive versus conservative glycemic control in patients undergoing coronary artery bypass graft surgery: A protocol for systematic review of randomised controlled trials
Source: PLoS One. 2022 Oct 18;17(10):e0276228. doi: 10.1371/journal.pone.0276228 (PMC9578579; doi:10.1371/journal.pone.0276228)
Supplement: Attachment — Submitted filename: Peer review.docx [file pone.0276228.s007.docx]

**Peer review**

In this protocol, the authors tried to describe the objectives, eligibility criteria, and planned methodology of their systematic review that will assess the effect of different intraoperative glycemic control strategies on postoperative outcomes. Evidence shows that poor intraoperative glycemic control is a risk factor for postoperative morbidity and mortality. Different strategies are used for intraoperative control of blood sugar, but their effect on postoperative outcomes has been conflicting across studies. Therefore, this systematic review aims to examine this crucial question and, more specifically, to determine the effect intensive glycemic control of blood sugar has on postoperative outcomes versus conservative control. The title is interesting and the protocol has been written according to the PRISMA-P statement. However, there are several points in this protocol that require further clarification, as follows:

**Introduction:**

- The authors clearly have described the condition, how glycemic control during cardiac surgery might affect postoperative outcomes, and how different glycemic control strategies might work.
- The purpose of the study has been explained in a very general way. The authors should specify exactly what research questions this review ultimately answers.
- Place the point after the closing bracket of the citations.

**Methods:**

- The authors should describe some items of eligibility criteria more clearly and in detail:

1) Regarding the target population, will you include all patients undergoing CABG surgery and intraoperative glycemic control, regardless of their demographic characteristics (e.g., age), background, and past medical history of diabetes mellitus?
2) Regarding types of interventions, please explain clearly what range of blood sugar control you consider to define “intensive” and “conservative” control.
3) Please specify the comparison group more clearly. Will you only include studies that considered high levels of glycemic control target (conservative group) as the comparison group?
4) Since you discussed the eligibility criteria based on the PICOS in the 2.2 subsection, and the outcomes are also one of its items, there is no need to describe the outcomes in a separate section. Please leave the explanation of subsection 2.3, which is about the outcomes, below in subsection 2.2.
5) The authors should describe the report characteristics of studies to be included, such as years considered, language, and publication status. If relevant studies published in all languages are to be included, how to deal with articles that have a language different from the language of the authors?

- Study selection, data synthesis, and risk of bias assessment have been described according to the PRISMA-P statement.

**Other comments:**

- The authors should arrange the order of the abstract sections according to the main text (Introduction/background, Methods, Discussion).
- The manuscript has some typing mistakes and grammatical errors that need to be corrected
