# Supplementary material for: Intensive versus conservative glycemic control in patients undergoing coronary artery bypass graft surgery: A protocol for systematic review of randomised controlled trials
Source: PLoS One. 2022 Oct 18;17(10):e0276228. doi: 10.1371/journal.pone.0276228 (PMC9578579; doi:10.1371/journal.pone.0276228)
Supplement: Attachment — Submitted filename: Peer review. R1.docx [file pone.0276228.s009.docx]

**Peer review (Revision 1)**

Dear Editor,

The authors have satisfactorily addressed all comments, and the manuscript has improved. The protocol now contains all of the necessary details to conduct a standard systematic review study. I have nothing further to add.

Regards
