# Supplementary material for: Intensive versus conservative glycemic control in patients undergoing coronary artery bypass graft surgery: A protocol for systematic review of randomised controlled trials
Source: PLoS One. 2022 Oct 18;17(10):e0276228. doi: 10.1371/journal.pone.0276228 (PMC9578579; doi:10.1371/journal.pone.0276228)
Supplement: Attachment — Submitted filename: Response to Reviewers.docx [file pone.0276228.s010.docx]

Dear editor and reviewers,

Thanks for your letter and the reviewers’ comments concerning our manuscript entitled “Intensive versus conservative glycemic control in patients undergoing coronary artery bypass graft surgery: A protocol for systematic review of randomised controlled trials” (PONE-D-22-18985). The comments are very helpful and valuable for revising and improving our manuscript. We would like to thank you for allowing us to submit a revised copy of the manuscript, and we highly appreciate your time and consideration. We have read through those comments carefully and revised the manuscript accordingly. In addition to the response letter, a marked and clean version of our revised manuscript were uploaded based on the instructions provided in your letter. Revised portions are marked in red in the marked version of revised manuscript.

**Reviewer#1:**

**The authors have well revised the manuscript; however, I can still see some errors in writing, particularly in using the articles (i.e., the). As an example from the abstract, it should be written: "the intensive care unit (ICU), the incidence of postoperative myocardial infarction), the incidence of ....". Or in discussion of the Abstract "There is no certain evidence that which strategy of glycemic...." is better to change "There is no certain evidence to clarify which strategy of glycemic....". Please double check the manuscript for these issues.**

**Response:** Thank you for your valuable suggestions. Following the reviewer’s suggestions, we carefully corrected the mistakes and double checked the manuscript. And the corrections were marked in red in the marked version.

Once again, thank you very much for your efforts in reviewing our manuscript.

Your sincerely,

Meng Lv

Department of Anesthesiology, The First Affiliated Hospital of Shandong First Medical University & Shandong Provincial Qianfoshan Hospital, Ji'nan, Shandong Province, 250014, China.

Telephone: +8615169105373

Email: qylvmeng@163.com
